# Survival time after marked reduction in oral intake in terminally ill noncancer patients: A retrospective study

**DOI:** 10.1002/jgf2.290

**Published:** 2019-12-06

**Authors:** Takahiro Hosoi, Sachiko Ozone, Jun Hamano

**Affiliations:** ^1^ Faculty of Medicine Division of Clinical Medicine Department of General Medicine University of Tsukuba Tsukuba Ibaraki Japan; ^2^ Faculty of Medicine Kamisu Clinical Education and Training Center University of Tsukuba Kamisu Ibaraki Japan; ^3^ Faculty of Medicine Division of Clinical Medicine University of Tsukuba Tsukuba Ibaraki Japan

**Keywords:** elderly, geriatrics, palliative medicine

## Abstract

**Background:**

The prediction of short‐term survival is important for noncancer patients and their families. Although a markedly reduced oral intake by cancer patients suggests a poor prognosis, the survival times of noncancer patients after its onset remain unclear. We herein investigated the time from a marked reduction in oral intake to death in noncancer patients as well as factors associated with their subsequent survival.

**Methods:**

We conducted a retrospective medical record review of noncancer patients who died in our hospital between April 2017 and April 2018. We recorded the day when oral intake markedly decreased and the date of death. We extracted data on age, gender, the Charlson Comorbidities Index, mean daily fluid volume, laboratory test results, and vital signs converted to the Shock Index (SI). We used Cox's proportional hazards models to assess relationships between these factors and survival times after the onset of a markedly reduced oral intake.

**Results:**

We analyzed data from 44 noncancer patients. The median time from the onset of a markedly reduced oral intake to death was 16.5 days. Based on Cox's proportional hazards models, only SI ≧ 1.0 at the onset of a markedly reduced oral intake correlated with survival times (hazard ratio: 5.89, 95% confidence interval (CI): 1.71‐20.1, *P* = .005).

**Conclusion:**

Noncancer patients died a median of 16.5 days after the onset of a markedly reduced oral intake, and SI ≧1.0 correlated with subsequent survival times. These results will provide novel insights into the prognosis of noncancer patients at the end of life.

## Graphical abstract

Predicting the short‐term survival is important for noncancer patients and their families. We found that noncancer patients died a median of 15.0 days after their oral intake markedly decreased, and Shock Index was related to their subsequent survival time. Our results will provide new insights into the prognosis of noncancer patients at the end of life.

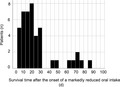

## 1 INTRODUCTION

Patients and their families facing life‐threatening illnesses are entitled to receive palliative care. Approximately half of all deaths worldwide are due to chronic diseases, and chronic, noncancer patients account for two‐thirds of deaths in Japan.^1^, ^2^ The palliative care needs of noncancer patients are becoming more important, but have not yet been sufficiently addressed.^3^

Care in the final phase of life is one of the essential elements of palliative care. According to a survey on the bereavement of cancer patients in the United States, losing patients unexpectedly caused depression and complex grief in their families.^4^ A nationwide survey of the bereaved family members of cancer patients in Japan also reported that meaningful communication between patients and family members in their last days of life was associated with improvements in psychiatric conditions such as depression and complex grief.^5^ An accurate diagnosis of impending death is also important for medical professionals to make important decisions such as calling precious family members in time, stopping aggressive treatment, and moving patients to private rooms.^6^, ^7^

Several signs of impending death in advanced cancer patients have recently been reported.^8^, ^9^ A marked reduction in oral intake, for example, is a weekly to daily prognostic factor, which is one of the components of prognostic tools such as the Palliative Prognostic Index (PPI).^10^ In addition to symptoms, many studies have been published on biomarkers in dying patients.^11^ Vital signs, which are routinely measured in daily practice, are also used to assess the prognosis of dying patients.^12^ The Shock Index (SI), defined as the ratio of heart rate to systolic blood pressure, has recently attracted attention for its relationship with short‐term survival times in terminal cancer patients.^13^ The development of prognostic tools using these objective and subjective data has enabled medical professionals to relatively accurately estimate the end of life and provide appropriate care for terminal cancer patients and their families.^14^, ^15^, ^16^

The final stage of life is also important for noncancer patients; however, few studies have investigated the symptoms and signs that suggest imminent death. Although a marked reduction in oral intake is considered to be a poor prognostic factor in nonmalignant patients, limited information is currently available on its onset. The survival time after a reduced oral intake differs among individual patients; however, the factors related to this duration remain unknown. The identification of these factors may contribute to more accurate predictions of the prognosis of noncancer patients and also improve their quality of care and death. The purpose of the present study was to examine the development of a markedly reduced oral intake and investigate factors related to subsequent survival times in noncancer patients.

## 2 METHODS

### 2.1 Study design and participants

The present study was a retrospective, medical record review of noncancer patients (age ≥ 20 years) admitted to the general internal medicine ward of Kamisu Saiseikai Hospital in Ibaraki, Japan. This hospital is one of the regional core hospitals that serves a region of approximately 250 000 individuals, and patients with acute illnesses are mainly hospitalized. Based on previous studies,^11^, ^14^, ^17^ we employed platelet counts, albumin (Alb), blood urea nitrogen (BUN), and SI as factors related to survival times after a markedly reduced oral intake and estimated the number of cases required for a multivariable analysis as 40‐50. Since there were approximately 4‐5 appropriate subjects for the present study per month in our hospital, we set the observation period to 12 months. We included patients who died between April 2017 and April 2018. Patients who were diagnosed with cancer, who received artificial nutrition (tube feeding or central venous nutrition), or who were on a ventilator during hospitalization were excluded. As described in the Data collection section below, since the assessment of a reduced oral intake required a minimum of 3 days, we excluded patients who died within 3 days of their admission. The Institutional Review Board of Kamisu Saiseikai Hospital waived the need for informed consent from study patients. Information on the present study was presented on a notice board in the hospital to provide an opportunity for study subjects to refuse. All procedures performed were in accordance with the 1964 Helsinki Declaration.

### 2.2 Data collection

Baseline characteristics, including age, gender, diagnosis on admission, and the presence of comorbidities using the Charlson Comorbidities Index (CCI), were recorded for all study patients.^18^ Based on previous studies,^8^, ^10^, ^19^ we consulted with several experts of General Internal Medicine and palliative care to establish a definition for “the onset of a markedly reduced oral intake.” Since we estimated that a patient's oral intake varied day to day and gradually decreased, we ultimately reached a definition using the following steps: (a) We investigated a patient's oral intake from the day of admission and identified the day at which daily oral intake decreased to less than a few mouthfuls. (b) After that day, if the patient was unable to eat more than half of the meal per day for two or more days, that day was defined as the onset of a markedly reduced oral intake. When there were multiple candidate days, we defined the first candidate date seen from the day of admission as the onset of a markedly reduced oral intake. We then recorded the number of days until the date of death. The mean daily fluid volume from the day of becoming anorexic to death was also investigated. Laboratory test results from the closest day to the onset of a reduced oral intake were investigated, including white blood cell counts, lymphocyte counts, platelet counts, albumin, blood urea nitrogen, creatinine, aspartate aminotransferase, alanine transaminase, alkaline phosphatase, total bilirubin, lactate dehydrogenase, sodium (Na), potassium (K), and CRP. Vital signs, including systolic and diastolic blood pressure, body temperature, and heart rate, were routinely documented. Vital signs measured on the onset day of a markedly reduced oral intake were recorded. We also converted systolic blood pressure and pulse to SI. Based on previous studies,^13^, ^20^, ^21^ SI was classified into 3 categories.

### 2.3 Statistical analysis

We summarized baseline characteristics using descriptive statistics. We excluded patients who were unable to eat on the day of admission to avoid the underestimation of results. The median time of the onset of a markedly reduced oral intake was estimated using the Kaplan‐Meier method. Univariate Cox's proportional hazards models were used to assess whether baseline demographics and laboratory test results were related to survival times after a poor oral intake in all study patients. All variables with a *P* < .05 in univariable analyses were tested in multivariable models to evaluate whether they were associated with survival times after the reduction in oral intake. We used the forced entry method to adjust for all confounding variables. All data were analyzed using SPSS, version 25 (IBM Japan, Ltd.). In all analyses, a *P* < .05 was considered to be significant.

## 3 RESULTS

**Figure 1:**
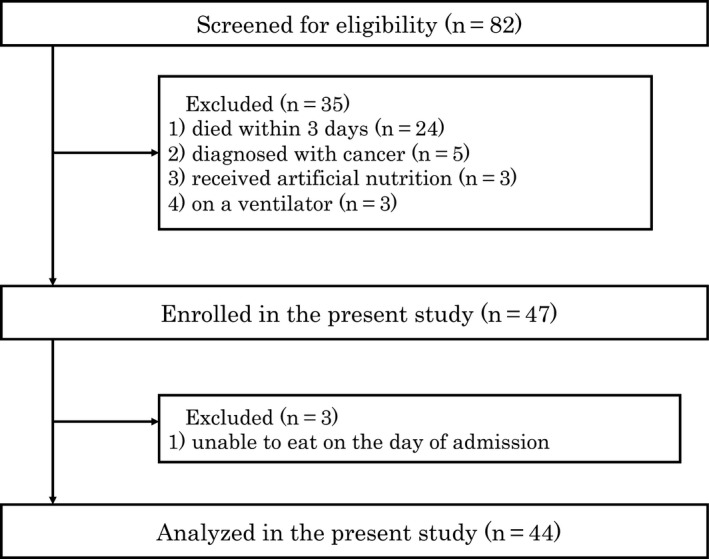
Flowchart of the study design. Patients who died within 3 d, were diagnosed with cancer, received artificial nutrition, or were on a ventilator were excluded

**Figure 2:**
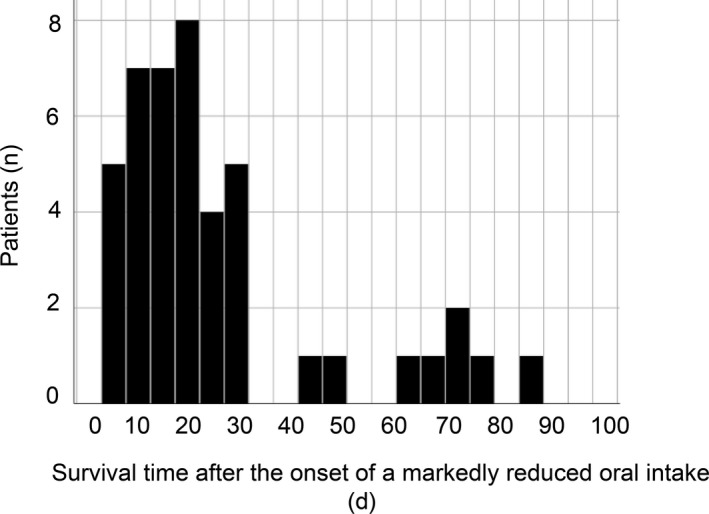
Survival time after the onset of a marked reduction in oral intake. The median number of days before death after the onset of a marked reduction in oral intake was 16.5

Eighty‐two patients died during the study period. We excluded 35 patients who met our exclusion criteria, and, thus, 47 patients were examined. We excluded 3 patients who were unable to eat less than a few mouthfuls on the day of admission and ultimately analyzed 44 patients (Figure 1). The baseline characteristics of the study patients are summarized in Table 1. The mean age of subjects was 80.3 years old, and 54.6% were male. Respiratory diseases were the most common disease at admission among all patients. The median number of days from death after the onset of a markedly reduced oral intake was 16.5 (Figure 2).

**Table 1:** Demographics and clinical characteristics of study subjects

Baseline characteristics	All patients (n = 44)
Age (mean ± SD)	80.3 ± 9.2
Male (%)	24 (54.6)
Disease at admission, n (%)	
Cerebrovascular disease	2 (4.5)
Cardiovascular disease	4 (9.1)
Respiratory disease	18 (40.9)
Liver disease	3 (6.8)
Renal disease	2 (4.5)
Others	15 (34.1)
Charlson Comorbidity Index (mean ± SD)	2.8 ± 1.8
Mean daily fluid volume (L)	1.32 ± 1.73
Length of hospital stay (median, interquartile range)	36.7 [6.2, 55.0]
Survival time after becoming anorexic (median, interquartile range)	16.5 [8.3, 26.5]
Laboratory data of the analyzed patients (mean ± SD)	
White blood cell count (×10^3^/μL)	9.77 ± 6.40
Lymphocyte count (%)	12.6 ± 11.7
Platelet count (×10^4^/μL)	18.1 ± 10.9
C reactive protein (mg/dL)	6.3 ± 8.0
Albumin (g/dL)	2.7 ± 0.5
Aspartate aminotransferase (/mL)	40.1 ± 39.8
Alanine transaminase (U/mL)	37.6 ± 95.2
Blood urea nitrogen (mg/dL)	41.1 ± 31.6
Creatinine (mg/dL)	1.7 ± 1.7
Lactate dehydrogenase (U/mL)	392.4 ± 725.6
Sodium (mEq/L)	139.2 ± 10.3
Total bilirubin (mg/dL)	1.8 ± 3.8
Alkaline phosphatase (U/mL)	315.4 ± 221.3
Vital signs at the onset of a reduced oral intake	
Systolic blood pressure (mmHg)	118.8 ± 25.8
Diastolic blood pressure (mmHg)	69.7 ± 16.7
Body temperature (°C)	36.8 ± 0.85
Heart rate (bpm)	86.8 ± 21.6
Shock index (%)	
SI < 0.7	20 (45.4)
0.7 ≦ SI < 1.0	19 (43.2)
SI ≧ 1.0	5 (11.4)

The results of univariable and multivariable Cox's proportional hazards analyses are shown in Table 2. Univariable analyses confirmed that platelet counts, LDH, heart rate, and SI ≥ 1.0 correlated with survival times after the onset of anorexia. As a result of an examination of multiple collinearity between possible predictors, we found a strong correlation between HR and SI. We finally adopted only SI, which strongly correlated with survival times. Age, which was considered to be important in clinical settings and previous studies,^9^, ^10^, ^14^ and platelet counts, LDH, and SI were forced into the final model. Multivariable analyses confirmed that only SI ≥ 1.0 correlated with survival times (hazard ratio: 5.89, 95% confidence interval (CI): 1.71‐20.1, *P* = .005).

**Table 2:** Results of univariable and multivariable cox's proportional hazards analyses for factors related to survival times after the development of anorexia

Variables	Univariable analyses (All patients, n = 44)	
	Hazard ratio (95% CI)	*P*‐value
Age	1.00 (0.97‐1.03)	.90
Female (vs male)	0.55 (0.29‐1.01)	.06
Mean daily fluid volume (L)	1.00 (0.80‐1.24)	.97
Charlson Comorbidity Index	0.92 (0.76‐1.11)	.36
Laboratory results		
White blood cell count (×10^3^/μL)	0.99 (0.94‐1.04)	.68
Lymphocyte count (%)	1.03 (0.99‐1.06)	.11
Platelet count (×10^4^/μL)	0.97 (0.94‐0.99)	.03
C reactive protein (mg/dL)	0.97 (0.93‐1.01)	.19
Albumin (g/dL)	1.13 (0.61‐2.10)	.69
Aspartate aminotransferase (/mL)	1.00 (0.99‐1.01)	.40
Alanine transaminase (U/mL)	1.00 (0.99‐1.00)	.82
Blood urea nitrogen (mg/dL)	0.99 (0.99‐1.01)	.45
Creatinine (mg/dL)	0.87 (0.72‐1.06)	.17
Lactate dehydrogenase (U/mL)	1.00 (1.00‐1.00)	.009
Sodium (mEq/L)	0.99 (0.96‐1.02)	.42
Total bilirubin (mg/dL)	1.04 (0.96‐1.14)	.33
Alkaline phosphatase (U/mL)	0.99 (0.99‐1.00)	.56
Vital signs at the onset of a reduced oral intake		
Shock index(SI)		
SI < 0.7	1 (reference)	
0.7 ≦ SI < 1.0	1.88 (0.930‐3.80)	.079
SI ≧ 1.0	9.46 (3.06‐29.2)	<.001
Systolic blood pressure (mmHg)	1.00 (098‐1.01)	.51
Diastolic blood pressure (mmHg)	1.00 (0.99‐1.02)	.66
Body temperature (°C)	1.06 (0.73‐1.55)	.76
Heart rate (bpm)	1.02 (1.01‐1.04)	.009

Variables	Multivariable analyses	
	Hazard ratio (95% CI)	*P*‐value
Age	1.02 (0.98‐1.05)	.34
Lactate dehydrogenase	1.00 (1.00‐1.00)	.17
Platelet count	0.98 (0.95‐1.01)	.102
Shock index		
SI < 0.7	1 (reference)	
0.7 ≦ SI < 1.0	1.69 (0.81‐3.5)	.16
SI ≧ 1.0	5.89 (1.71‐20.1)	.005

Abbreviation: CI, Confidence interval.

## 4 DISCUSSION

The present results suggest that noncancer patients died a median of 16.5 days after the onset of a marked reduction in oral intake, and SI ≥ 1.0 at this onset was related to subsequent survival times.

The most important result of the present study was that the time from the onset of a markedly reduced oral intake and death was a median of 16.5 days. Thus, a poor oral intake in noncancer patients may develop earlier than in advanced cancer patients. In a prospective study on 357 end‐stage cancer patients to investigate physical signs associated with death, the median onset of anorexia was 7.5 days from death (95% CI: 5.0‐9.5).^22^ A previous study found that almost all terminally ill advanced cancer patients developed anorexia, which appeared to have a negative impact on their quality of end of life.^23^, ^24^ Many factors contribute to a poor oral intake in cancer patients, including cachexia, digestive system issues, and other physical symptoms (such as pain, dyspnea, and dysphagia). Since subjects in the present study were end‐stage patients with different disease states, as shown in Table 1, the causes of a poor oral intake are expected to be diverse.

The second important result of the present study was that SI may be a useful prognostic tool for short‐term survival times in noncancer patients after a reduction in oral intake. SI has been recognized as a poor prognostic factor in emergencies.^20^, ^21^ In a retrospective cohort study of patients with advanced cancer, the combination of SI ≥ 1.0 and a decreased level of consciousness was identified as a useful assessment tool for short‐term survival times.^13^ SI was demonstrated to be a sensitive indicator of left ventricular dysfunction, and a persistently high SI suggests an unstable hemodynamic state in critically ill patients.^25^ An increase in SI at the onset of a markedly reduced oral intake in noncancer patients indicates an unstable hemodynamic state. We considered this to be why SI was associated with the subsequent survival period.

There are several limitations to the present study. Since this was a retrospective medical record review, potential cofounding factors such as patient symptoms (including comatose, edema, and dyspnea) and other laboratory data as described by Reid VL et al^11^ may not have been considered. Furthermore, the present study was conducted at a single center. In addition, the population of the present study only included acutely ill patients who received many drugs, such as antihypertensive medications, antiarrhythmic drugs, antibiotics, and steroids, which may have affected their vital signs and survival times. However, the results obtained reflected clinical settings because this study was conducted at the internal medicine ward of a general acute care hospital in Japan. Further studies need to be conducted on other patient populations, such as those in home care settings and nursing homes. Another limitation is that the definition of “the onset of a markedly reduced oral intake” was original to present study and may be complicated. However, we were able to standardize the assessment of the onset using this definition and oral intake by patients gradually decreased from the onset date. Regardless of these limitations, the present results will contribute to clarifying the prognosis of noncancer patients.

## 5 CONCLUSION

We found that noncancer patients died a median of 16.5 days after the onset of a markedly reduced oral intake, and SI was related to subsequent survival times. The present results will provide novel insights into the prognosis of noncancer patients at the end of life.

## CONFLICTS OF INTEREST

The authors have stated explicitly that there are no conflicts of interest in connection with this article.
